# Supplementary material for: Comparative Studies of the Uptake and Internalization Pathways of Different Lipid Nano-Systems Intended for Brain Delivery
Source: Pharmaceutics. 2023 Aug 3;15(8):2082. doi: 10.3390/pharmaceutics15082082 (PMC10458318; doi:10.3390/pharmaceutics15082082)
Supplement: Supplementary file 1 [file pharmaceutics-15-02082-s001.zip › pharmaceutics-2458896-supplementary.pdf]

pharmaceutics

MDPI

Supplementary Material: Comparative Studies of the Uptake and Internalization Pathways of Different Lipid Nano-systems Intended for Brain Delivery

Ljubica Mihailova, Dushko Shalabalija, Andreas Zimmer, Nikola Geskovski, Petre Makreski, Marija Petrushevska, Maja Simonoska Crcarevska and Marija Glavash Dodov

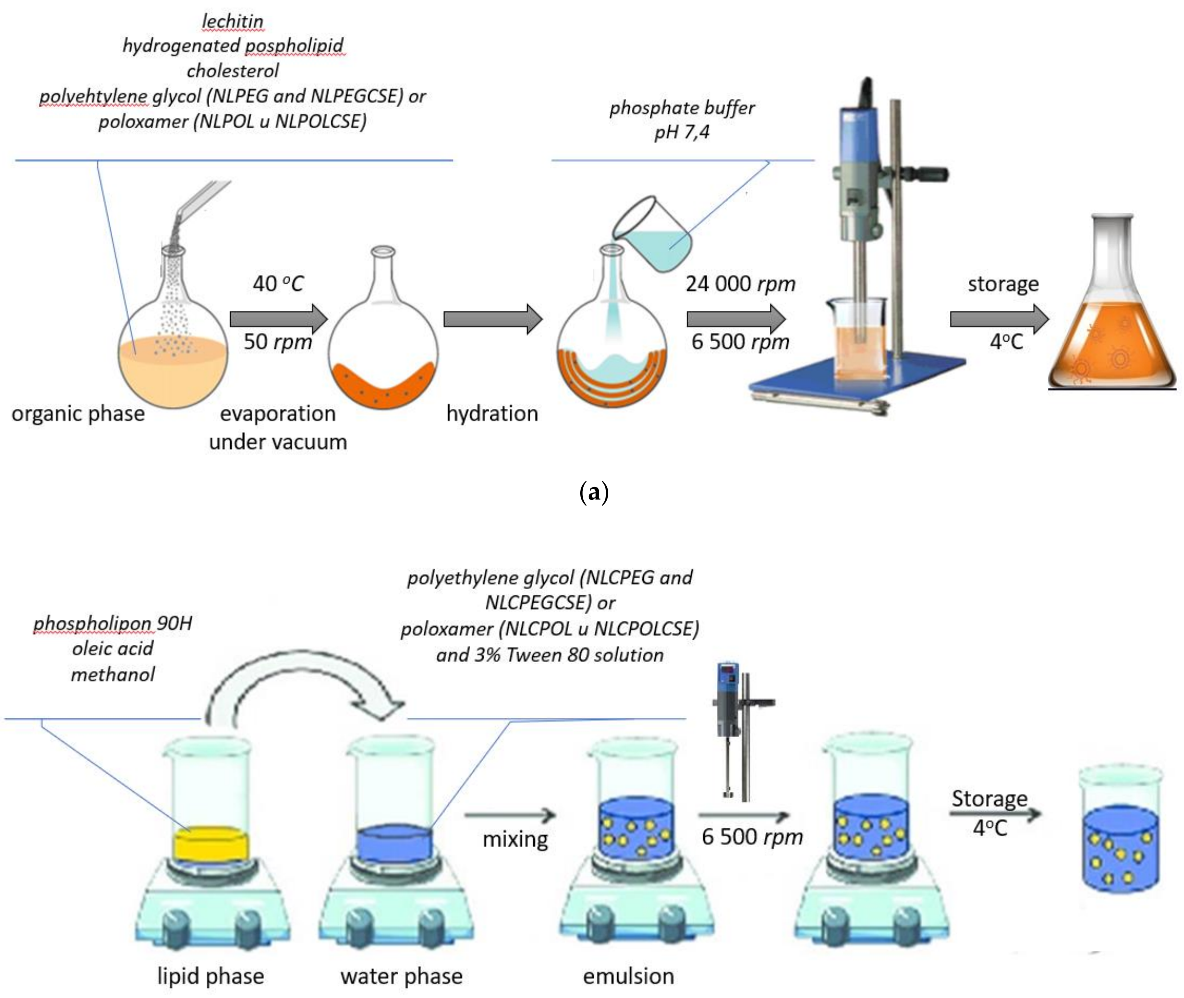

(a)

(b)

Figure S1. Schematic view of the preparation of (**a**) nanoliposomes and (**b**) nanostructured lipid carriers.

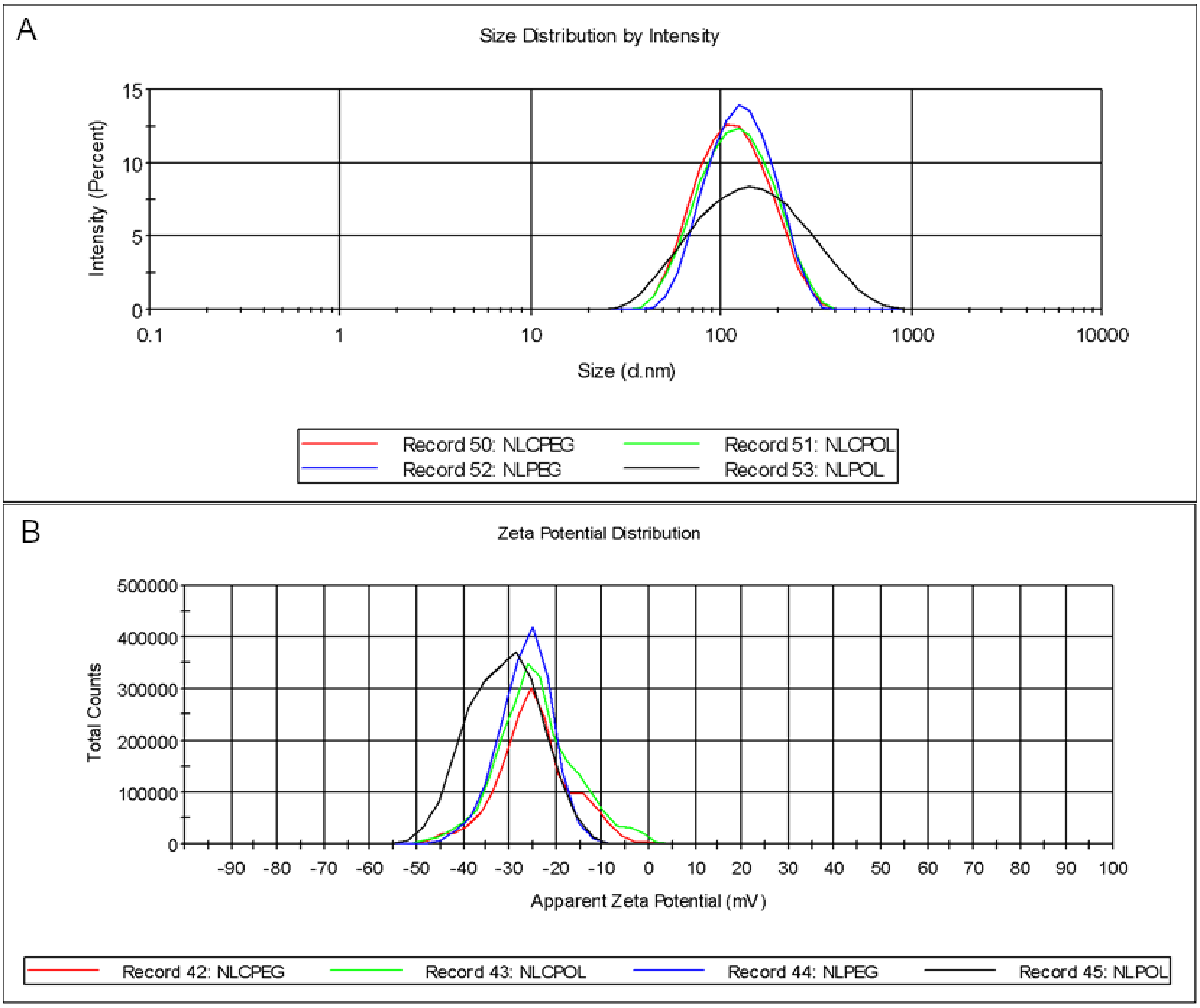

Figure S2. Prepared formulations characterized by (**A**) particle size and particle size distribution; (**B**) zeta potential.

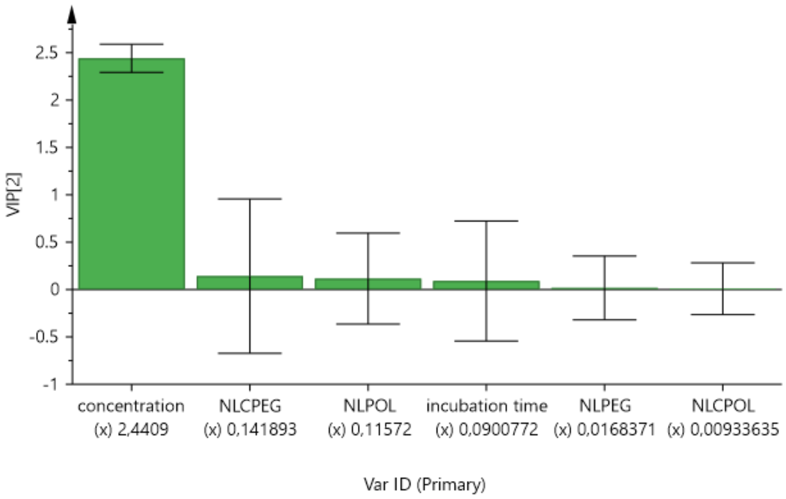

Figure S3. VIP plot of significant factors in cell viability experiments.

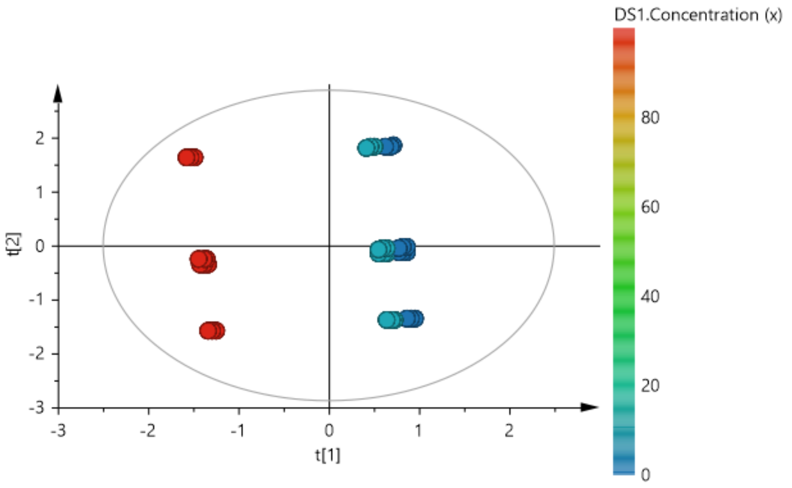

Figure S4. Scatter plot of analyzed sample scores colored by incubation time.

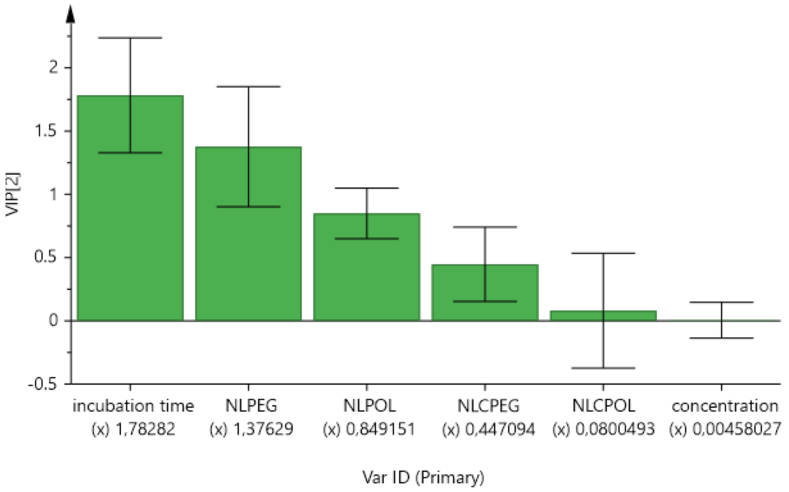

Figure S5. VIP plot of significant factors in cell cytotoxicity experiments.

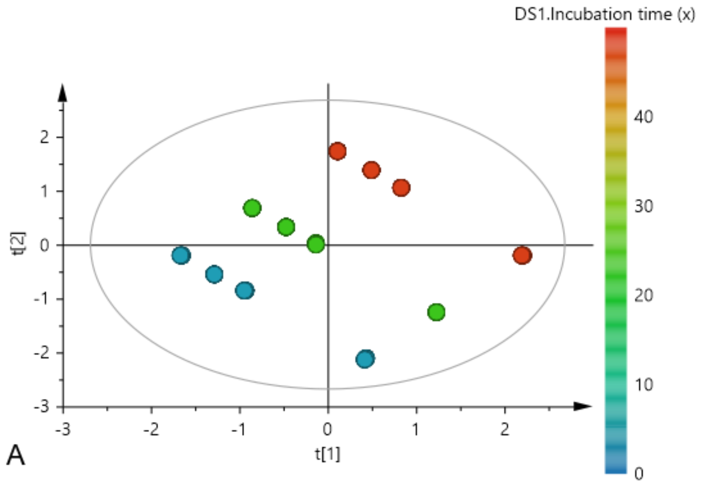

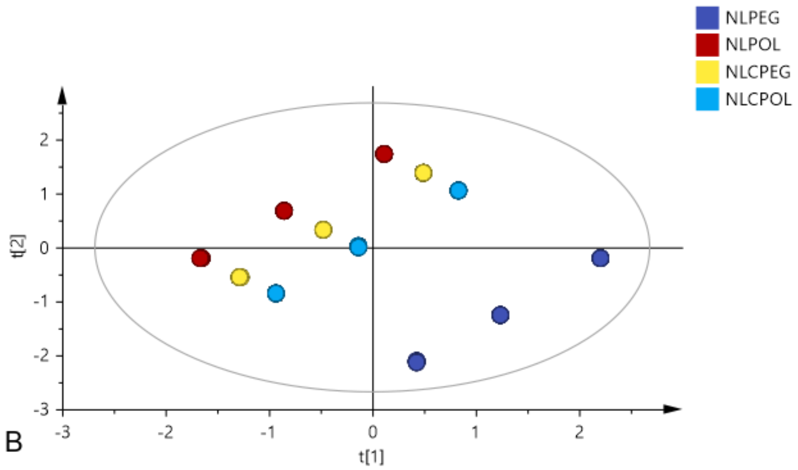

Figure S6. Scatter plot of analyzed sample scores colored by: (**A**) time incubation; (**B**) formulation.

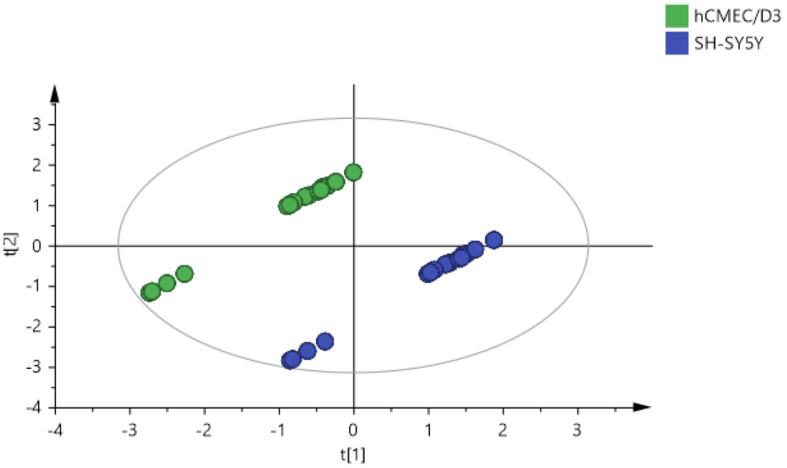

Figure S7. Scatter plot of analyzed results colored by cell line type.

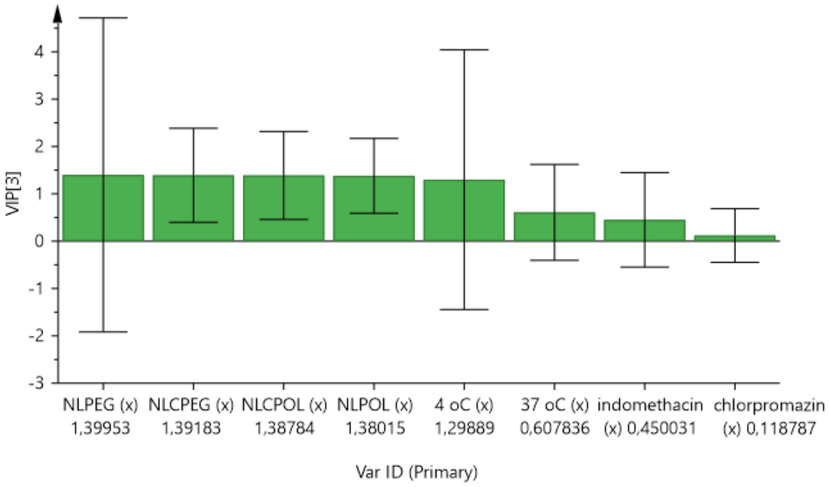

Figure S8. VIP plot of significant factors affecting the cell uptake of nano formulations in hCMEC/D3 cell line.

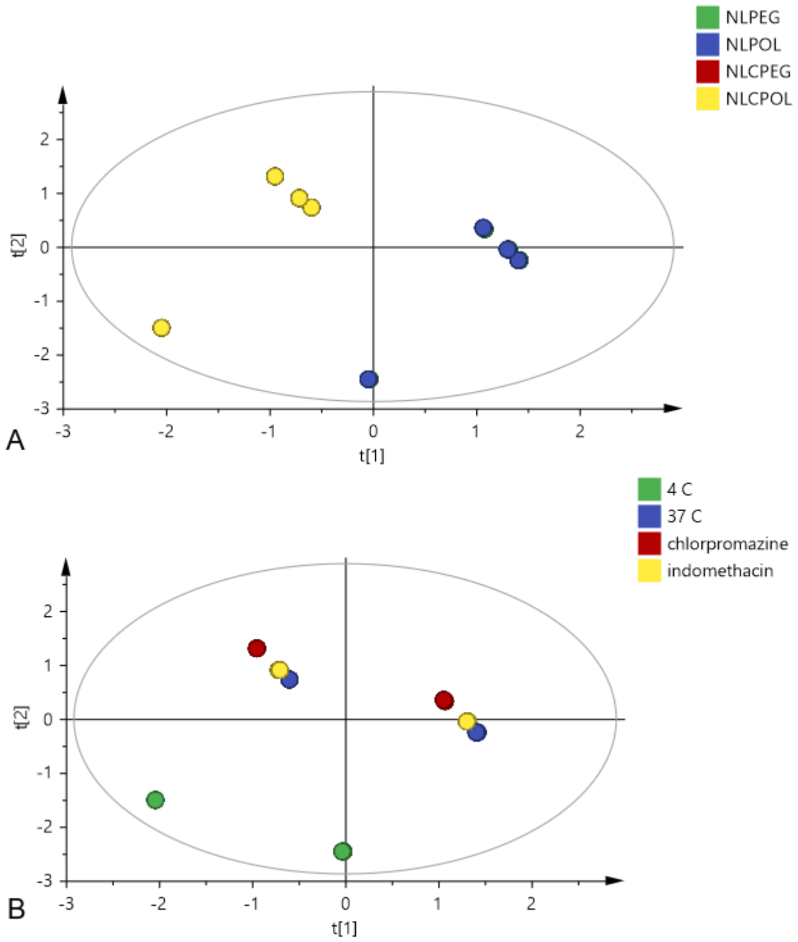

Figure S9. Scatter plot of analyzed sample scores on hCMEC/D3 cell line colored by (**A**) type of formulation; (**B**) experimental conditions.

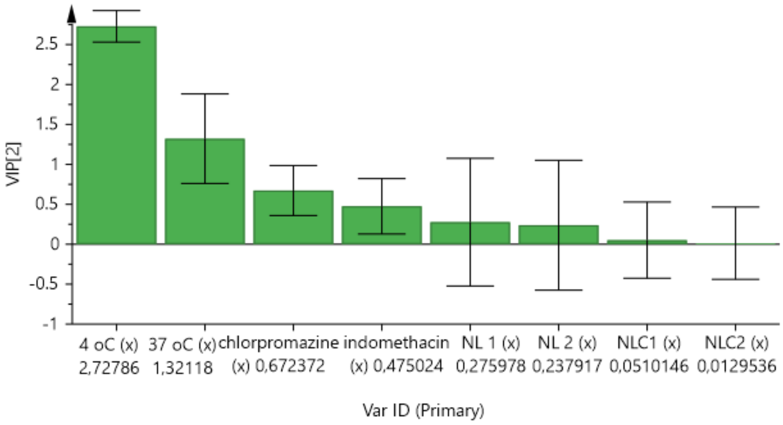

Figure S10. VIP plot of significant factors affecting the cell uptake of nano formulations in SH-SY5Y cell line.

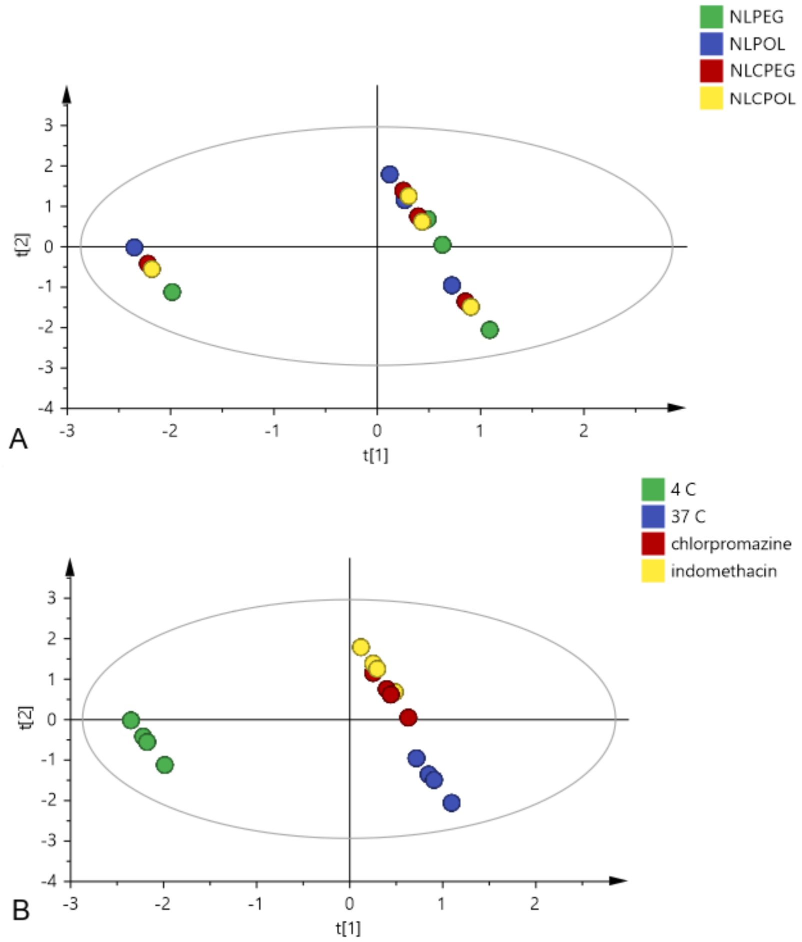

Figure S11. Scatter plot of analyzed sample scores on SH-SY5Y cell line colored by (**A**) type of formulation; (**B**) experimental conditions.
